# High Prevalence of *Rickettsia raoultii* and Associated Pathogens in Canine Ticks, South Korea

**DOI:** 10.3201/eid2610.191649

**Published:** 2020-10

**Authors:** Min-Goo Seo, Oh-Deog Kwon, Dongmi Kwak

**Affiliations:** Animal and Plant Quarantine Agency, Gimcheon, South Korea (M.-G. Seo);; Kyungpook National University, Daegu, South Korea (O.-D. Kwon, D. Kwak)

**Keywords:** ticks, phylogeny, Ehrlichia, Rickettsia, Theileria, vector-borne infections, dogs, bacteria, South Korea

## Abstract

We studied the prevalence of tickborne pathogens in canine ticks, South Korea, during 2010–2015. Results revealed a high prevalence of the emerging pathogen *Rickettsia raoultii*. Dog ticks may be maintenance hosts for tickborne pathogens, suggesting the need to continually evaluate the potential public health threat posed by *R. raoultii–*infected ticks.

Ticks are responsible for mechanical damage to animal blood vessels and skin and are known to transmit a wide range of bacteria, viruses, and protozoa, causing severe infections in animals and humans (*1*). Most defined Rickettsiales are considered zoonotic emerging or reemerging pathogens; some can cause severe human illnesses, including anaplasmosis, rickettsioses, scrub typhus, and ehrlichiosis (*2*). Determining the ecology of local tick species and recognizing the tickborne pathogens they carry are of paramount public health importance. Our study assessed risk factors for and the prevalence and co-infectivity of several tickborne pathogens in ticks collected from dogs in South Korea. 

*Rickettsia* spp. are emerging or reemerging pathogens with public health relevance; 1 species, *R. raoultii*, causes human tickborne lymphadenitis in many countries in Europe (*3*). Of note, *R. raoultii* had not been detected in humans, animals, or vectors in South Korea until recently, but it now appears to be endemic in ticks infesting dogs. We collected a total of 980 ticks in central (n = 442) and southern (n = 538) South Korea from 102 dogs during 2010–2015. We used both morphological and molecular methods (Appendix) to identify the tick species, which included *Haemaphysalis longicornis*, *H. flava*, and *Ixodes nipponensis*, then sorted them into 364 pools (1–7 ticks per pool) by dog, identified tick species, and developmental stage (larva, nymph, and adult). 

Our findings are consistent with the results of a previous study from South Korea, in which *H. longicornis* ticks were found in 201 (48.9%), *Haemaphysalis* spp. ticks in 130 (31.6%), *H. flava* ticks in 71 (17.3%), and *I. nipponensis* ticks in 7 (1.7%) of 411 dogs (*4*). A previous study of *H. longicornis* tick prevalence proposed that, rather than rodents as previously thought, larger mammals, including dogs, might be the hosts for this tick species (*5*). Additional surveys are needed to assess the natural hosts of *H. longicornis* ticks. 

Several tickborne pathogens were then screened by using primer sets specific to each pathogen (Appendix). The 16S rRNA genes of *R. raoultii* were found in 149 (40.9%), *R. monacensis* in 1 (0.3%), and *Candidatus* Rickettsia principis in 2 (0.6%) of 364 tick pools (Figure; Appendix Table 1). *R. raoultii* was detected in 100 nymph and 49 adult *H. longicornis* ticks in South Korea. *R. raoultii*–positive ticks were collected from 25 (24.5%) of 102 dogs, a relatively high proportion of those observed in this study. 

**Figure:**
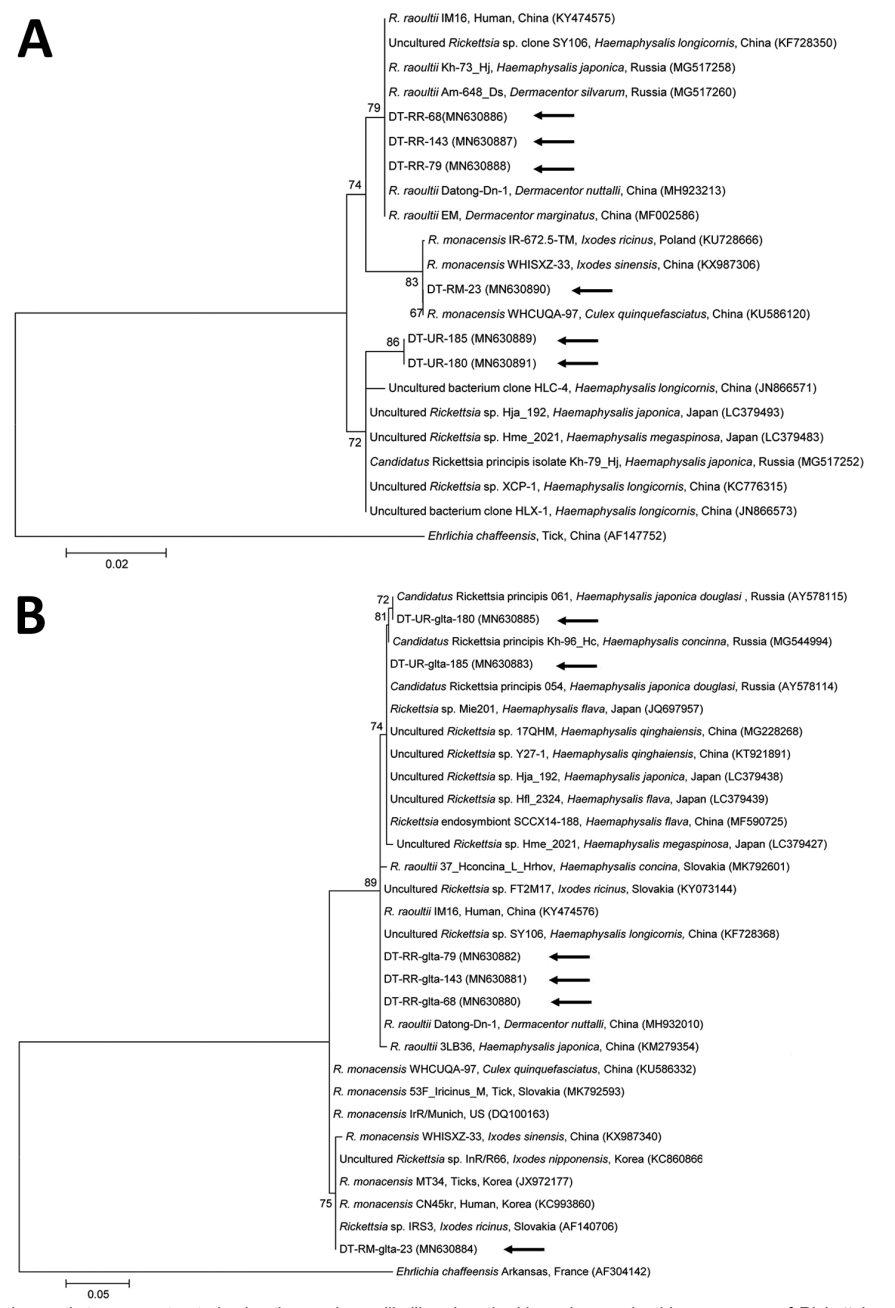
Phylogenetic trees constructed using the maximum-likelihood method based on nucleotide sequences of *Rickettsia* spp. from canine ticks, South Korea (black arrows), and reference sequences. A) 16S rRNA; (B) *glt*A. *Ehrlichia chaffeensis* sequences were used as outgroups. GenBank accession numbers for reference sequences are shown with the sequence name. Branch numbers indicate bootstrap support (1,000 replicates). Scale bar indicates phylogenetic distance.

*R. monacensis* causes spotted fever–like disease and has been found in multiple hard tick species in several European countries (*2*). It was detected in 16 (55.2%) of 29 pools of *I. nipponensis* ticks from small mammals in South Korea (*6*). In this study, however, *R. monacensis* was found in only 1 (0.3%) of 364 tick pools, in an adult *I. nipponensis* tick. One spotted fever group rickettsiae with *Candidatu*s status was also identified in ticks in this study; *Candidatus* R. principis was identified in 2 (3.0%) of 67 *H. japonica douglasii* ticks in Russia in 2006 (*7*). In this study, *Candidatus* R. principis (0.6%) was detected in 1 *H. longicornis* nymph and 1 *H. flava* nymph. Additional tickborne pathogens were detected (Appendix Table 1, Figures 1, 2): the *E. canis* 16S rRNA gene was identified in 1 *H. longicornis* nymph (0.3%), and the *T. luwenshuni* 18S rRNA gene was identified in 20 *H. longicornis* nymphs (10.9%) and 24 *H. longicornis* adults (26.1%). No other tickborne pathogens were detected in this study. 

Increased seasonal tick populations and activity in the summer and autumn impact the transmission of tickborne pathogens (*8*). In this study, we collected ticks from May to September, and found that tick abundance and distribution patterns were similar to those in a previous study in South Korea (*8*), which showed that both ticks and tickborne pathogens were more prevalent in southern regions and during the summer. South Korea is also steadily shifting to a subtropical climate due to global warming (*9*), which may influence this seasonal effect, as well. In another previous study in South Korea (*4*), ticks were collected from stray or pet dogs, but no ticks were found on military working dogs. These military dogs received routine veterinary care for preventive ectoparasite treatments. Therefore, tick prevention measures should be effective in endemic areas with known tick seasons, when infestations are higher. 

Our findings indicate the zoonotic potential of dog ticks in South Korea. Physicians and public health officers therefore need to be aware of the high potential and clinical complexity of infection with *R. raoultii* and other tickborne pathogens in order to confirm suitable testing and treatment needs in endemic areas (*10*). Therefore, we strongly recommend continuous evaluation of the potential public health threat posed by infected ticks to humans in South Korea. A better understanding of local tick species, including *H. longicornis*, and a more thorough characterization of TBP agents, such as *R. raoultii*, are critical. 

Appendix Additional methods and results for high prevalence of *Rickettsia raoultii* and associated pathogens in canine ticks, South Korea.

## References

[1] Dantas-Torres F, Chomel BB, Otranto D. Ticks and tick-borne diseases: a One Health perspective. Trends Parasitol. 2012;28:437–46. 10.1016/j.pt.2012.07.003 22902521

[2] Raoult D, Parola P, editors. Rickettsial diseases. New York: Informa Healthcare; 2007.

[3] Oteo JA, Portillo A. Tick-borne rickettsioses in Europe. Ticks Tick Borne Dis. 2012;3:271–8. 10.1016/j.ttbdis.2012.10.035 23177355

[4] Choe HC, Fudge M, Sames WJ, Robbins RG, InYong L, Chevalier NA, et al. Tick surveillance of dogs in the Republic of Korea. Syst Appl Acarol. 2011;16:215–22. 10.11158/saa.16.3.5

[5] Zheng H, Yu Z, Zhou L, Yang X, Liu J. Seasonal abundance and activity of the hard tick *Haemaphysalis longicornis* (Acari: Ixodidae) in North China. Exp Appl Acarol. 2012;56:133–41. 10.1007/s10493-011-9505-x 22113778

[6] Lee KM, Choi YJ, Shin SH, Choi MK, Song HJ, Kim HC, et al. Spotted fever group rickettsia closely related to *Rickettsia monacensis* isolated from ticks in South Jeolla province, Korea. Microbiol Immunol. 2013;57:487–95. 23621111 10.1111/1348-0421.12062

[7] Mediannikov O, Sidelnikov Y, Ivanov L, Fournier PE, Tarasevich I, Raoult D. Far eastern tick-borne rickettsiosis: identification of two new cases and tick vector. Ann N Y Acad Sci. 2006;1078:80–8. 10.1196/annals.1374.010 17114683

[8] Im JH, Baek J, Durey A, Kwon HY, Chung MH, Lee JS. Current status of tick-borne diseases in South Korea. Vector Borne Zoonotic Dis. 2019;19:225–33. 10.1089/vbz.2018.2298 30328790

[9] Seo MG, Lee SH, VanBik D, Ouh IO, Yun SH, Choi E, et al. Detection and genotyping of *Coxiella burnetii* and *Coxiella*-like bacteria in horses in South Korea. PLoS One. 2016;11:e0156710. 10.1371/journal.pone.0156710 27244230 PMC4886966

[10] Li H, Zhang PH, Huang Y, Du J, Cui N, Yang ZD, et al. Isolation and identification of *Rickettsia raoultii* in human cases: a surveillance study in 3 medical centers in China. Clin Infect Dis. 2018;66:1109–15. 10.1093/cid/cix917 29069294

