# Supplementary material for: High Prevalence of Rickettsia raoultii and Associated Pathogens in Canine Ticks, South Korea
Source: Emerg Infect Dis. 2020 Oct;26(10):2530–2. doi: 10.3201/eid2610.191649 (PMC7510725; doi:10.3201/eid2610.191649)
Supplement: Appendix — Additional methods and results for high prevalence of Rickettsia raoultii and associated pathogens in canine ticks, South Korea. [file 19-1649-Techapp-s1.pdf]

Article DOI: https://doi.org/10.3201/eid2610.191649

High Prevalence of *Rickettsia raoultii* and Associated Pathogens in Canine Ticks, South Korea

Appendix

Supplemental Methods

Tick Collection and Species Identification

A total of 980 ticks were collected from stray dogs in animal shelters located in central South Korea: Chungbuk (130 ticks from 14 dogs), Chungnam (145 ticks from 15 dogs), and Gyeongbuk (167 ticks from 17 dogs) and southern South Korea: Jeonbuk (165 ticks from 17 dogs), Jeonnam (180 ticks from 19 dogs), and Gyeongnam (193 ticks from 20 dogs). None of the infested dogs showed clinical symptoms of tickborne pathogens (TBPs). To note, several dogs had skin redness around the tick bites. Five to 10 ticks per dog were collected from a total of 102 dogs. We collected ticks from dogs from their head, neck, abdomen areas, and mouth parts with fine forceps and gently removed attached ticks. We then stored ticks in vials containing 70% ethanol. Ticks were first identified to the species level and classified morphologically into several developmental stages (*1*). Subsequently, different tick species were pooled as follows: per dog, identified species, and developmental stages (larva, nymph, and adult) into 364 tick pools, with 1 to 7 ticks per pool.

Tick and TBP Detection

Genomic DNA was extracted from each tick pool sample (*n* = 364 pools) using a commercial DNeasy Blood & Tissue Kit (Qiagen, https://www.qiagen.com/us) according to the manufacturer’s instructions. Extracted DNA was then stored at −20°C until further use. The AccuPower HotStart PCR Premix kit (Bioneer, https://eng.bioneer.com) was used for PCR amplification. Molecular identification of tick species was conducted by amplifying the mitochondrial cytochrome c oxidase subunit I (COI) gene using specific primers (*2*). Several

TBPs were then screened by using specific primer sets for each pathogen. For example, rickettsial infections (*Anaplasma*, *Ehrlichia*, and *Rickettsia*) were initially assessed using a commercial AccuPower Rickettsiales 3-Plex PCR Kit (Bioneer) to detect 16S rRNA. Positive samples were then amplified further for species identification. For *Rickettsia* spp., positive samples were confirmed by PCR targeting the citrate synthase gene (*glt*A) (*3*). The 5S (*rrf*)–23S (*rrl*) intergenic spacer gene of *Borrelia* spp. and the outer surface protein A gene fragment of *Borrelia burgdorferi* sensu lato was amplified by nested PCR (nPCR) (*4*). Multiple primer sets were used to amplify the *Coxiella* 16S rRNA gene, including *C. burnetii* and *Coxiella*-like bacteria (*5*). Piroplasm infections (*Babesia* and *Theileria*) were first screened using a commercial AccuPower Rickettsiales 2-Plex PCR Kit (Bioneer) to detect piroplasm 18S rRNA. The positive samples were then re-amplified by PCR using primers designed from the common sequence of the 18S rRNA genes of several *Babesia* species (*6*). Nested primer sets were used to amplify the internal transcribed spacer region sequence of *Bartonella* spp (*7*). The S segment of SFTSV was amplified by nPCR (*8*). Appendix Table 2 describes the primers and amplification conditions used for TBP detection in this study.

DNA Cloning

Amplicons of 364 pooled tick and 197 TBP-positive samples were purified using the QIAquick Gel Extraction Kit (Qiagen), ligated into pGEM-T Easy vectors (Promega, https://www.promega.com), transformed into *Escherichia coli* DH5α-competent cells (Thermo Fisher Scientific, https://www.thermofisher.com), and incubated at 37°C overnight. Plasmid DNA extraction was performed using a plasmid miniprep kit (Qiagen) according to the manufacturer’s instructions.

DNA Sequencing and Phylogenetic Analysis

Recombinant clones were selected and sent to Macrogen (https://www.macrogen.com/en/main/index.php) for sequencing. The sequences were analyzed and aligned using the multiple sequence alignment program CLUSTAL Omega (v. 1.2.1), and the alignment was edited with BioEdit (v. 7.2.5). Phylogenetic analysis was performed using MEGA (v. 6.0) based on the maximum likelihood method with the Kimura 2-parameter distance

model. The aligned sequences were analyzed using a similarity matrix. The stability of the trees was estimated by bootstrap analysis using 1,000 replicates.

Statistical Analysis

GraphPad Prism (v. 5.04; GraphPad Software Inc., https://www.graphpad.com) was used for statistical analyses. The Chi-square test was performed to analyze significant differences among pathogens for each tick stage, and a value of $p < 0.05$ was considered statistically significant.

Supplemental Results

Tick Identification

Tick species were molecularly identified using primers for the COI gene (expected size 710 bp) to avoid potential mistakes in morphological identification. Three species were identified by morphological and molecular characteristics, and both methods showed congruent results. Furthermore, the nucleotide sequences from 8 representative ticks based on developmental stage and collected region were assessed for data analysis.

The 3 groups of ticks shared close genetic relationships with *H. longicornis* (98.0–100% nucleotide identity), *H. flava* (98.8–100% nucleotide identity), and *I. nipponensis* (98.9–100% nucleotide identity). A phylogenetic tree was assembled based on the COI genes of several ticks deposited in GenBank, and the ticks collected in this study were classified into 3 clades related to 3 species (Appendix Figure 3): *H. longicornis* (76.9%, 280/364 pools), *H. flava* (14.6%, 53/364 pools), and *I. nipponensis* (8.5%, 31/364 pools) (Appendix Table 1).

TBP Identification

In total, 69.6% (195/280) of *H. longicornis* ticks, including 66.7% (122/183) of nymphs and 79.3% (73/92) of adults, were PCR-positive for at least 1 TBP (Appendix Table 1). TBPs were significantly more abundant ($p = 0.0003$) in the adult stage compared to other developmental stages in *H. longicornis*. *R. raoultii* was the most abundant TBP in *H. longicornis*: 54.6% (100/183) of nymphs and 53.3% (49/92) of adults were PCR-positive. *T. luwenshuni* ($p = 0.0031$) was significantly more abundant in the adult stage compared to other stages in *H. longicornis*.

In *H. longicornis,* TBPs were detected in varying proportions in different geographical areas of South Korea: In the nymph stage, 53.8% (50/93) were detected in the central area and 80% (72/90) in the southern area; in the adult stage, 74.5% (35/47) were detected in the central area and 84.4% (38/45) in the southern area. TBPs were significantly more abundant in the adult stage of both central ($p = 0.0136$) and southern ($p = 0.002$) areas compared to the other stages in *H. longicornis*. *T. luwenshuni* from the southern area ($p = 0.0398$) was significantly more abundant in the adult stage compared to the other stages in *H. longicornis*. *R. raoultii* from the southern area ($p = 0.049$) was significantly more abundant in the nymph stage compared to the other stages in *H. longicornis*.

Among the positive samples, additional *glt*A gene analysis revealed that the ticks were positive for *R. raoultii* (43/364 pools, 11.8%), *R. monacensis* (1/364 pools, 0.3%), and *Candidatus* Rickettsia principis (2/364 pools, 0.6%). This was an expected result as comparisons of similarity values suggested that *glt*A is less conserved than the 16S rRNA gene in rickettsiae (*9*). The average rate of sequence change in *glt*A was quicker than the average rate of sequence change in the 16S rRNA gene. The *glt*A sequences may be valuable in uncovering close relationships.

R. raoultii-positive ticks were collected from dogs (24.5%, 25/102) from central South Korea: Chungbuk (2), Chungnam (3), and Gyeongbuk (6), and southern South Korea: (Jeonbuk (4), Jeonnam (4), and Gyeongnam (6). Eleven (3.0%) ticks were coinfected with *T. luwenshuni* and *R. raoultii*, while 1 (0.3%) tick was coinfected with *E. canis, T. luwenshuni,* and *R. raoultii.*

Molecular and Phylogenetic Analyses

H. longicornis COI gene sequences from this study showed 98.1–100% nucleotide identity with known *H. longicornis* COI gene sequences, consistent with the results of a phylogenetic analysis that classified *H. longicornis* into 2 groups with a neighborly relationship (Appendix Figure 3). *H. flava* COI gene sequences from this study showed 98.7–99.8% nucleotide identity and *I. nipponensis* COI gene sequences showed 98.5–99.5% nucleotide identity with known COI gene sequences (Appendix Figure 3).

Phylogenetic analyses showed that *E. canis* 16S rRNA nucleotide sequences (Appendix Figure 1) and *T. luwenshuni* 18S rRNA nucleotide sequences (Appendix Figure 2) were clustered with previously GenBank documented sequences.

The 1 *E. canis* sequence found in the present study shared 99.4–100% identity with the 16S rRNA gene in previously reported *E. canis* isolates. The 3 *T. luwenshuni* representative sequences from the present study shared 99.8–100% identity with the 18S rRNA gene. They also shared 96.6–99.8% identity with the 18S rRNA gene previously reported in *T. luwenshuni* isolates.

The 3 *R. raoultii* representative sequences from the present study shared 100% identity with 16S rRNA and 99.7–100% identity with *glt*A genes. They also shared 99.4–99.7% identity with 16S rRNA and 98.1–99.7% identity with *glt*A genes in previously reported *R. raoultii* isolates. The 1 *R. monacensis* sequence found in the present study shared 99.1–99.6% identity with 16S rRNA and 99.1–100% identity with *glt*A genes in previously reported *R. monacensis* isolates. The 2 sequences of *Candidatus* R. principis genes found in the present study shared 100% identity with 16S rRNA and 98.7% identity with *glt*A genes. They also shared 98.5–98.9% identity with 16S rRNA and 99.2–100% identity with the *glt*A genes in previously reported *Candidatus* R. principis isolates.

The representative sequences in this study were submitted to GenBank. Accession numbers are MN630872 (*I. nipponensis*), MN630873 (*H. flava*), MN630874–MN630879 (*H. longicornis*), MN630892 (*E. canis*), MN626388–MN626390 (*T. luwenshuni*), and MN630880–MN630891 (*Rickettsia* spp.).

References

1. Barker SC, Walker AR. Ticks of Australia. The species that infest domestic animals and humans. Zootaxa. 2014;3816:1–144. PubMed https://doi.org/10.11646/zootaxa.3816.1.1

2. Folmer O, Black M, Hoeh W, Lutz R, Vrijenhoek R. DNA primers for amplification of mitochondrial cytochrome c oxidase subunit I from diverse metazoan invertebrates. Mol Mar Biol Biotechnol. 1994;3:294–9. PubMed

3. Reis C, Cote M, Paul RE, Bonnet S. Questing ticks in suburban forest are infected by at least six tick-borne pathogens. Vector Borne Zoonotic Dis. 2011;11:907–16. PubMed https://doi.org/10.1089/vbz.2010.0103

4. VanBik D, Lee SH, Seo MG, Jeon BR, Goo YK, Park SJ, et al. *Borrelia* species detected in ticks feeding on wild Korean water deer (*Hydropotes inermis*) using molecular and genotypic analyses. J Med Entomol. 2017;54:1397–402. PubMed https://doi.org/10.1093/jme/tjx106

5. Seo MG, Lee SH, VanBik D, Ouh IO, Yun SH, Choi E, et al. Detection and genotyping of *Coxiella burnetii* and *Coxiella*-like bacteria in horses in South Korea. PLoS One. 2016;11:e0156710. PubMed https://doi.org/10.1371/journal.pone.0156710

6. Casati S, Sager H, Gern L, Piffaretti JC. Presence of potentially pathogenic *Babesia* sp. for human in *Ixodes ricinus* in Switzerland. Ann Agric Environ Med. 2006;13:65–70. PubMed

7. Ko S, Kim SJ, Kang JG, Won S, Lee H, Shin NS, et al. Molecular detection of *Bartonella grahamii* and *B. schoenbuchensis*-related species in Korean water deer (*Hydropotes inermis argyropus*). Vector Borne Zoonotic Dis. 2013;13:415–8. PubMed https://doi.org/10.1089/vbz.2012.1105

8. Yoshikawa T, Fukushi S, Tani H, Fukuma A, Taniguchi S, Toda S, et al. Sensitive and specific PCR systems for detection of both Chinese and Japanese severe fever with thrombocytopenia syndrome virus strains and prediction of patient survival based on viral load. J Clin Microbiol. 2014;52:3325–33. PubMed https://doi.org/10.1128/JCM.00742-14

9. Roux V, Rydkina E, Eremeeva M, Raoult D. Citrate synthase gene comparison, a new tool for phylogenetic analysis, and its application for the rickettsiae. Int J Syst Bacteriol. 1997;47:252–61. PubMed https://doi.org/10.1099/00207713-47-2-252

Appendix Table 1. Prevalence of tickborne pathogens detected by PCR in ticks from dogs in South Korea, 2010–2015

Species	Region	Stage	No. of tick pools	No. positive (%)					
				E. canis (16S rRNA)	*T. luwenshuni* (18S rRNA)	*R. raoultii* (16S rRNA)	*R. monacensis* (16S rRNA)	*Candidatus* Rickettsia principis (16S rRNA)	Total
Haemaphysalis longicornis	Central	Larva	2	0	0	0	0	0	0
		Nymph	93	0	9 (9.7)	41 (44.1)	0	0	50 (53.8)
		Adult	47	0	11 (23.4)	24 (51.1)	0	0	35 (74.5)*
	Southern	Larva	3	0	0	0	0	0	0
		Nymph	90	1 (1.1)	11 (12.2)	59 (65.6)*	0	1 (1.1)	72 (80.0)
		Adult	45	0	13 (28.9)*	25 (55.6)	0	0	38 (84.4)*
	Subtotal	Larva	5	0	0	0	0	0	0
		Nymph	183	1 (0.6)	20 (10.9)	100 (54.6)	0	1 (0.6)	122 (66.7)
		Adult	92	0	24 (26.1)*	49 (53.3)	0	0	73 (79.3)*
Haemaphysalis flava	Central	Nymph	18	0	0	0	0	0	0
		Adult	10	0	0	0	0	0	0
	Southern	Nymph	12	0	0	0	0	1 (8.3)	1 (8.3)
		Adult	13	0	0	0	0	0	0
	Subtotal	Nymph	30	0	0	0	0	1 (3.3)	1 (3.3)
		Adult	23	0	0	0	0	0	0
Ixodes nipponensis	Central	Nymph	8	0	0	0	0	0	0
		Adult	6	0	0	0	0	0	0
	Southern	Nymph	10	0	0	0	0	0	0
		Adult	7	0	0	0	1 (14.3)	0	1 (14.3)
	Subtotal	Nymph	18	0	0	0	0	0	0
		Adult	13	0	0	0	1 (7.7)	0	1 (7.7)
		Total	364	1 (0.3)	44 (12.1)	149 (40.9)	1 (0.3)	2 (0.6)	197 (54.1)

*Designates significant differences in prevalence ($p < 0.05$) among the different stages.

Appendix Table 2. Primers used for the detection of tickborne pathogens in ticks from dogs.

Organism	Gene	Primer	Sequence 5' to 3'	Size (bp)	Amplification condition	Reference
Invertebrate	Mitochondrial cytochrome c oxidase subunit I	LCO1490	GGTCAACAAATCATAAAGATATTGG	710	95°C/5 min; 35 cycles: 95°C/60 s, 40°C/60 s, 72°C/30 s; 72°C/10 min	(*2*)
		HC02198	TAAACTTCAGGGTGACCAAAAAATCA			
Anaplasma spp.	16S rRNA	†		429	95°C/5 min; 45 cycles: 95°C/30 s, 59°C/30 s, 72°C/30 s; 72°C/10 min	Commercial AccuPower Rickettsiales 3-Plex PCR Kit (Bioneer)
Ehrlichia spp.	16S rRNA	†		340	95°C/5 min; 45 cycles: 95°C/30 s, 59°C/30 s, 72°C/30 s; 72°C/10 min	Commercial AccuPower Rickettsiales 3-Plex PCR Kit (Bioneer)
Rickettsia spp.	16S rRNA	†		252	95°C/5 min; 45 cycles: 95°C/30 s, 59°C/30 s, 72°C/30 s; 72°C/10 min	Commercial AccuPower Rickettsiales 3-Plex PCR Kit (Bioneer)
Rickettsia spp.	*glt*A	Rsfg877	GGGGGCCTGCTCACGGCGG	380	95°C/10 min; 35 cycles: 95°C/60 s, 51°C/60 s, 72°C/60 s; 72°C/10 min	(*3*)
		Rsfg1258	ATTGCAAAAAGTACAGTGAACA			
Borrelia spp.	5S–23S rRNA	N1	GAGCTTAAAGGAACTTCTGATAA	561	95°C/5 min; 35 cycles: 95°C/45 s, 54°C/45 s, 72°C/60 s; 72°C/5 min	(*4*)
		C1c	TTTGTACTGTTATTGTGTCTT			
		N2	ATGGWTCTGGAAYRCTYGAAG	533	95°C/5 min; 35 cycles: 95°C/45 s, 50°C/45 s, 72°C/60 s; 72°C/5 min	
		C2c	CTTARAGTAACWGTTCCTTCT			
Coxiella spp.	16S rRNA	Cox16SF1	CGTAGGAATCTACCTTRTAGWGG	1321–1429	93°C/3 min; 30 cycles: 93°C/30 s, 56°C/30 s, 72°C/60 s; 72°C/5 min	(*5*)
		Cox16SR2	GCCTACCCGCTTCTGGTACAATT			
		Cox16SF2	TGAGAACTAGCTGTTGGRRAGT	624–627	93°C/3 min; 30 cycles: 93°C/30 s, 56°C/30 s, 72°C/60 s; 72°C/5 min	
		Cox16SR2	GCCTACCCGCTTCTGGTACAATT			
Bartonella spp.	ITS-1	QHVE-OF	TTCAGATGATGATCCCAAGC	736	94°C/10 min; 35 cycles: 94°C/60 s, 55°C/60 s, 72°C/120 s; 72°C/5 min	(*7*)
		QHVE-OR	AACATGTCTGAATATATCTTC			
		QHVE-IF	CCGGAGGGCTTGTAGCTCAG	484		

Organism	Gene	Primer	Sequence 5' to 3'	Size (bp)	Amplification condition	Reference
		QHVE-IR	CACAATTTCAATAGAAC			
SFTVS	S segment	NP-2F	CATCATTGTCTTTGCCCTGA	461	50°C/30 min; 95°C/15 min; 40 cycles: 95°C/20 s, 52°C/40 s, 72°C/30 s; 72°C/5 min	(*8*)
		NP-2R	AGAAGACAGAGTTCACAGCA			
		N2F	AAYAAGATCGTCAAGGCATCA	346	25 cycles: 94°C/20 s, 55°C/40 s, 72°C/30 s	
		N2R	TAGTCTTGGTGAAGGCATCTT			
Babesia spp. and *Theileria* spp.	18S rRNA	†		676	95°C/5 min; 35 cycles: 95°C/30 s, 59°C/30 s, 72°C/30 s; 72°C/5 min	Commercial AccuPower *Babesia* and *Theileria* PCR Kit (Bioneer)
Babesia spp. and *Theileria* spp.	18S rRNA	BJ1	GTCTTGTAATTGGAATGATGG	452	95°C/5 min; 40 cycles: 94°C/30 s, 54°C/30 s, 72°C/40 s; 72°C/5 min	(*6*)
		BN2	TAGTTTATGGTTAGGACTACG			

*SFTVS, Severe fever with thrombocytopenia syndrome virus
*Commercial PCR kits were used for the detection of these pathogens.

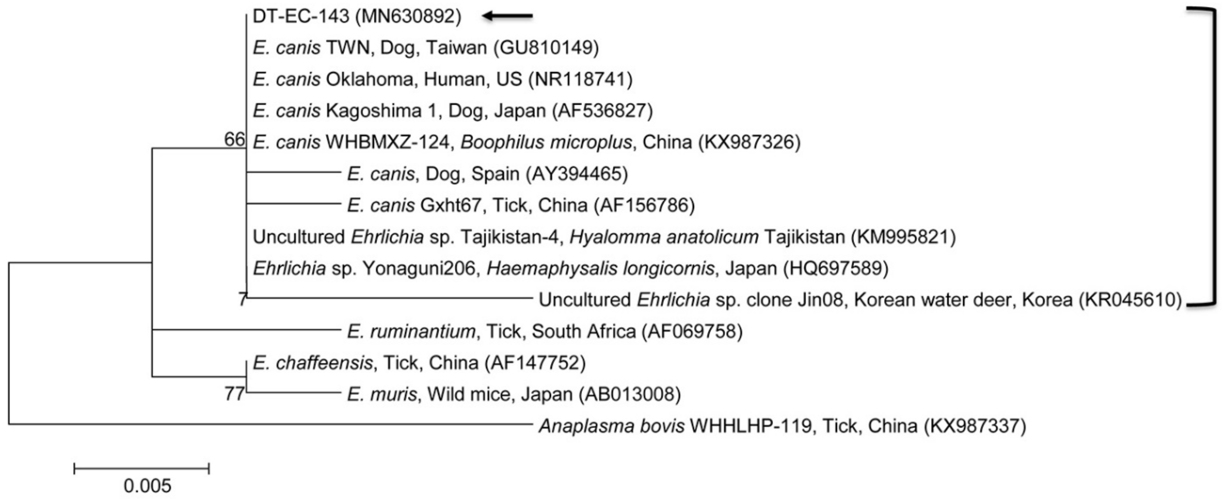

Appendix Figure 1. Phylogenetic tree constructed using the maximum likelihood method and based on *Ehrlichia canis* 16S rRNA nucleotide sequences. *Anaplasma bovis* was used as the outgroup. Black arrows indicate sequences analyzed in this study. GenBank accession numbers for other sequences are shown with the sequence name. Branch numbers indicate bootstrap support (1,000 replicates). Scale bar indicates phylogenetic distance.

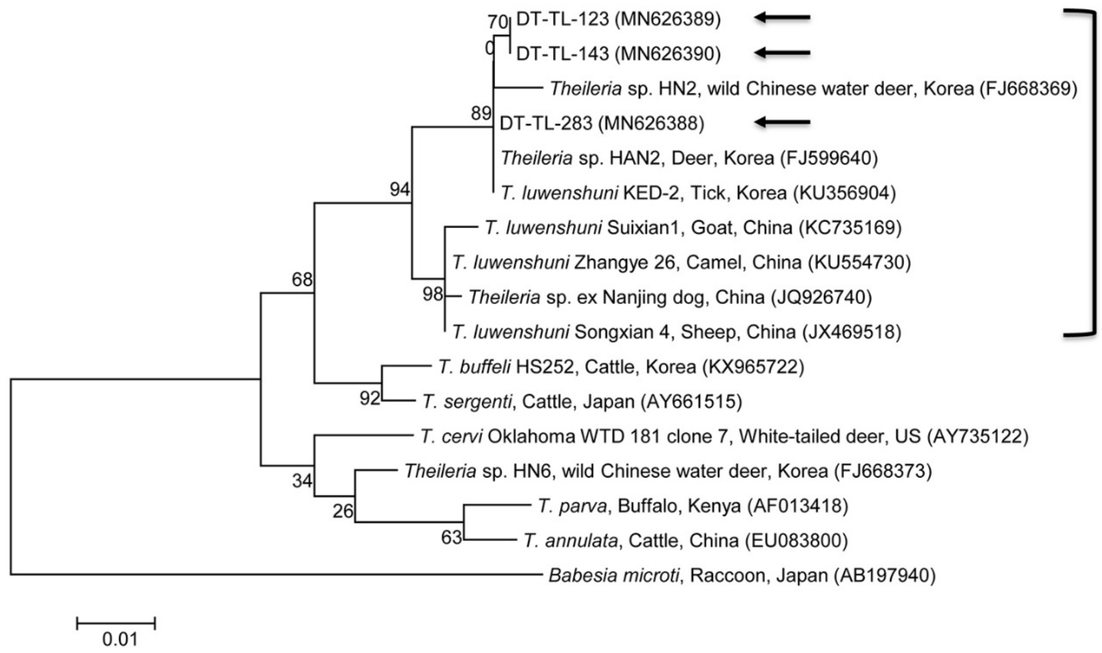

Appendix Figure 2. Phylogenetic tree constructed using the maximum likelihood method and based on *Theileria luwenshuni* 18S rRNA nucleotide sequences. *Babesia microti* was used as the outgroup. Black arrows indicate sequences analyzed in this study. GenBank accession numbers for other sequences are shown with the sequence name. Branch numbers indicate bootstrap support (1,000 replicates). Scale bar indicates phylogenetic distance.

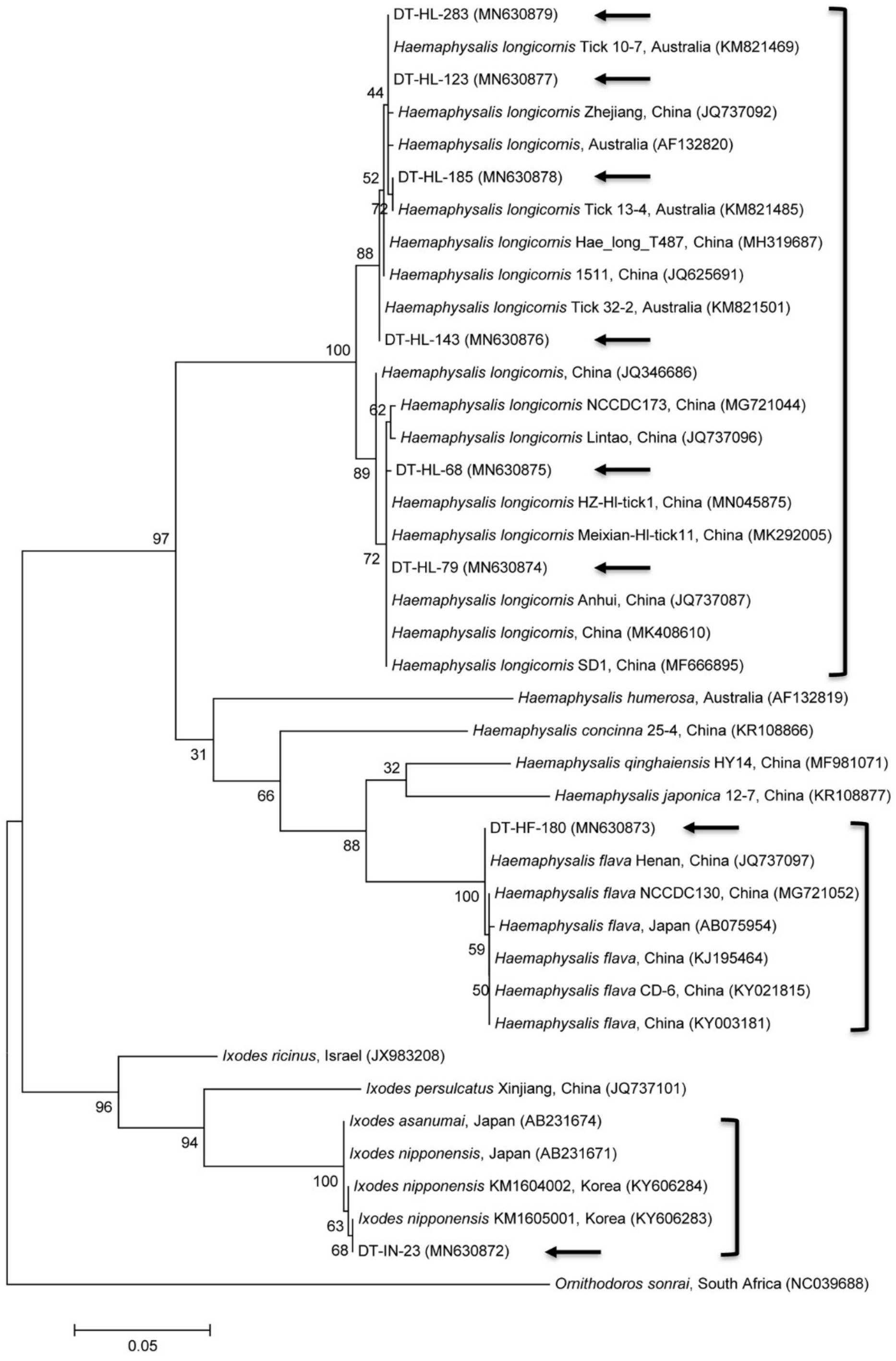

Appendix Figure 3. Molecular identification of ticks based on phylogenetic analysis using the maximum likelihood method with the mitochondrial cytochrome c oxidase subunit I gene. *Ornithodoros sonrai* was used as the outgroup. Black arrows indicate sequences analyzed in this study. GenBank accession numbers for other sequences are shown with the sequence name. Branch numbers indicate bootstrap support (1,000 replicates). Scale bar indicates phylogenetic distance.
